# Word2Vec inversion and traditional text classifiers for phenotyping lupus

**DOI:** 10.1186/s12911-017-0518-1

**Published:** 2017-08-22

**Authors:** Clayton A. Turner, Alexander D. Jacobs, Cassios K. Marques, James C. Oates, Diane L. Kamen, Paul E. Anderson, Jihad S. Obeid

**Affiliations:** 1 Department of Computer Science, College of Charleston, 66 George Street, Charleston, 29424 USA; 2 Department of Public Health Sciences, Medical University of South Carolina, 135 Cannon Street, Charleston, 29425 USA

**Keywords:** Natural language processing, Machine learning, Systemic lupus erythematosus

## Abstract

**Background:**

Identifying patients with certain clinical criteria based on manual chart review of doctors’ notes is a daunting task given the massive amounts of text notes in the electronic health records (EHR). This task can be automated using text classifiers based on Natural Language Processing (NLP) techniques along with pattern recognition machine learning (ML) algorithms. The aim of this research is to evaluate the performance of traditional classifiers for identifying patients with Systemic Lupus Erythematosus (SLE) in comparison with a newer Bayesian word vector method.

**Methods:**

We obtained clinical notes for patients with SLE diagnosis along with controls from the Rheumatology Clinic (662 total patients). Sparse bag-of-words (BOWs) and Unified Medical Language System (UMLS) Concept Unique Identifiers (CUIs) matrices were produced using NLP pipelines. These matrices were subjected to several different NLP classifiers: neural networks, random forests, naïve Bayes, support vector machines, and Word2Vec inversion, a Bayesian inversion method. Performance was measured by calculating accuracy and area under the Receiver Operating Characteristic (ROC) curve (AUC) of a cross-validated (CV) set and a separate testing set.

**Results:**

We calculated the accuracy of the ICD-9 billing codes as a baseline to be 90.00% with an AUC of 0.900, the shallow neural network with CUIs to be 92.10% with an AUC of 0.970, the random forest with BOWs to be 95.25% with an AUC of 0.994, the random forest with CUIs to be 95.00% with an AUC of 0.979, and the Word2Vec inversion to be 90.03% with an AUC of 0.905.

**Conclusions:**

Our results suggest that a shallow neural network with CUIs and random forests with both CUIs and BOWs are the best classifiers for this lupus phenotyping task. The Word2Vec inversion method failed to significantly beat the ICD-9 code classification, but yielded promising results. This method does not require explicit features and is more adaptable to non-binary classification tasks. The Word2Vec inversion is hypothesized to become more powerful with access to more data. Therefore, currently, the shallow neural networks and random forests are the desirable classifiers.

## Background

Electronic health record (EHR) phenotyping patients still relies heavily on International Classification of Diseases, Ninth Revision (ICD-9) and ICD-10 billing codes. ICD codes, however, have been known to be prone to errors due to a variety of problems in the coding and billing workflows [1–3]. This is problematic as clinicians use varying synonyms and abbreviations for the same condition and these error rates have been reported to range from 17.1 to 76.9% [1].

Classically, departing from ICD-9 codes would necessitate the usage of natural language processing (NLP) in order to extract features suitable for machine learning (ML) from the clinical notes in the EHR. Generating these features is not a straightforward step in this process as overtraining on specific datasets’ features are common, confounding variables can be prevalent, and the importance of specific words or phrases need to be considered. The task of identifying patients with certain criteria based on clinical notes in EHR can be a daunting one given the massive amounts of text notes. This phenotype classification task can be automated using NLP techniques along with pattern recognition ML algorithms, referred to as an NLP classifier. The key to finding these patterns is translating plain text into quantifiable entities that could be used as features for ML. The aim of this research is to evaluate combinations of NLP technique; and ML algorithms alongside a newer inversion-based method which utilizes word vectors based on Word2Vec, which does not have the problem of feature generation [4].

The classic technique for transforming textual data into features is Bag-of-Words (BOWs) [5], which maintains word frequencies. BOWs, while proven to be powerful for classification, can result in large number of terms and hence features, and does not consider negation [6]. Another well documented method is to use Unified Medical Language System (UMLS) Concept Unique Identifiers (CUIs) with ML [7]. Using CUIs in place of BOWs has become increasingly popular, in medical contexts, for assimilating synonyms in order to better represent and annotate textual data [8]. CUIs, when generated through a pipeline such as the clinical Text Analysis and Knowledge Extraction System (cTAKES), are able to detect negation in order to more accurately engineer relevant features [9, 10]. A textual note saying a patient does not have lupus would result in the concept for lupus being deducted by a count of one. In this same context, BOWs would increase the count of “lupus” and “not” by one, creating a malformed feature set when compared to CUIs. Although CUI-based methods are a significant improvement over plain term frequency, they still do not consider the sequence of terms or concepts in a document. A relatively recent method seen in clinical NLP systems and pipelines is the utilization of word vectors [11, 12]. Vectorization of sentences is being used more frequently as part of natural language and ML tasks with techniques such as Word2Vec and sentence2vec. Often, Word2Vec is used to create word clusters by averaging word vectors into clusters, referred to as word averaging, in order to be used in ML algorithms [12]. A relatively new technique that uses these word vectors, derived from Word2Vec, breaks the classification process into an ensemble of sentence classifications for a larger document. This technique is known as Word2Vec inversion.

The goal of this paper is to apply these methods to the phenotyping of SLE from clinical notes, and evaluate the performance of the different approaches. We detail the process in which we engineered our data for each of these different NLP techniques with justifications for each of the techniques we selected. Following is the evaluation of why specific techniques outperformed others and strongly outperformed the various baselines.

In order to evaluate these different techniques, we acquired data from the Medical University of South Carolina (MUSC) Rheumatology Clinic to function as a use case: the data obtained is for classifying Systemic Lupus Erythematosus (SLE), where the controls largely include patients with other rheumatological diseases. Clinicians’ diagnosis of SLE here is through the satisfaction of the American College of Rheumatology (ACR) classification for SLE and the more recently published Systemic Lupus Collaborating Clinics (SLICC) classification criteria [13–15]. The utility here is that one can identify patients for recruitment into SLE research studies through the use of these criteria. Therefore, diagnosis of SLE relies on the patient’s history of symptoms, physical examination findings, and results of blood and urine testing. These findings are then documented in their EHR clinical notes. As predescribed, clinicians look for specific criteria when evaluating SLE to aid in diagnosis, particularly ACR criteria [13, 14] and SLICC classification criteria [15]. Classification of SLE requires fulfillment of at least four ACR criteria (Table 1). The SLICC criteria allow for lupus nephritis to be the sole clinical criterion in the presence of ANA or anti-dsDNA antibodies [15].

**Table 1:** 1997 Update of the 1982 ACR revised criteria for the classification of SLE [13, 14]

Short description
1. Malar rash
2. Discoid rash
3. Photosensitivity
4. Oral ulcers
5. Nonerosive arthritis
6. Pleuritis or Pericarditis
7. Renal disorder
8. Neurologic disorder
9. Hematologic disorder
10. Immunologic disorder
11. Positive antinuclear antibody

We begin by describing the type of data utilized for this experiment. We then describe the different natural language techniques in order to transform the data and the machine learning algorithms applied to the different forms of transformed data. After this we detail, analyze, and discuss our results from the classification task. Concluding, we compare the strengths and weaknesses between the algorithms.

## Methods

### EHR clinical notes

Following IRB approval, we acquired a dataset of 662 patients from the Rheumatology Clinic, 322 patients diagnosed with SLE and 340 controls. The diagnoses of all SLE patients in our data set were confirmed by trained rheumatologists. All SLE patients met 1997 classification criteria for SLE [13], while controls were confirmed as not meeting those criteria. These 662 patient records contain a total of 250,434 clinical notes. In order to remove noise introduced by notes that were not relevant to rheumatology, such as referrals for other problems or simple lab visits, we applied a filter to include only relevant clinical notes using the stem word “rheumatol”. A simple Python script was used to aggregate clinical notes for each patient into larger patient-centric documents. Bootstrap resampling, randomly selecting a sample to add to the pipeline from the class with less instances was used to achieve a balanced dataset with an equal number of patients and controls in both training and testing data sets [16]. One hundred patients were set aside to be used as an external testing data set. ICD-9 code data on these patients were used as a simple baseline classifier for lupus patients. At the institution of study, MUSC, ICD-9 codes are entered by the physician. The ICD-9 code for SLE is 710.0.

### Natural language processing

We used several NLP methods to transform the clinical notes into features for machine learning algorithms. In order to extract CUIs from the notes, we utilized cTAKES [9]. The cTAKES system is a pipeline composed of components and annotators, including the utilization of term frequency-inverse document frequency to identify and normalize CUIs. This pipeline is a bottom-up NLP system which starts with sentence boundary detection and tokenizing and works up to part-of-speech tagging and named entity recognition. In conjunction with cTAKES, we utilized the Yale cTAKES Extension for Document Classification (YTEX) which adds components to the cTAKES pipeline: the pipeline adds long-range negation detection and a database consumer which adds database-reading functionality to catalyze the process of note processing and UMLS concept extraction and annotation [7, 10]. The CTAKES/YTEX pipeline ends with an exporter which retrieves curated the CUIs.

#### Bag-of-Words

BOWs in its simplest form is an orderless representation of word frequencies in a document. In the context of this patient classification problem, the word counts from each note are combined for each patient and normalized into a term frequency matrix prior to classification. The Python natural language toolkit (nltk) and native Python String library [17, 18] were used for this step. Python’s String library was used to parse out punctuation. Stop words were removed using nltk. This was followed by stemming using nltk’s SnowballStemmer [17].

#### Concept unique identifiers

In order to extract CUIs from the notes, we utilized cTAKES [9]. The Yale cTAKES Extension (YTEX), was used to export cTAKES annotations into machine learning formats [10]. The end product is a CUI frequency feature matrix.

#### Word2Vec

Word2Vec is an NLP system that utilizes neural networks in order to create a distributed representation of words in a corpus [19]. While the BOW and CUI pipelines produce word frequency and CUI frequency for each document respectively, Word2Vec creates vectors for each word present in a document. These vectors have a smaller distance between them for related words, for example, Athens and Greece and pluralities or tense switches, such as alumnus and alumni or walking and walked [19]. In order to map words to vectors, Word2Vec uses an underlying shallow neural network in addition to techniques seen in deep learning tasks. This unsupervised task takes each individual sentence for a given corpus and, in the neural network, encodes the context of each of the words in the sentence, much like the deep learning autoencoders seen in restricted Boltzmann machines and Stacked Denoising Autoencoders [20, 21]. This is done through the usage of skip-grams, which calculates the probabilities of occurrence of words within a certain distance before and after a given word. Inter-relating these probabilities creates similar word vectors for those with higher probabilities.

### Data processing and machine learning

**Fig. 1:**
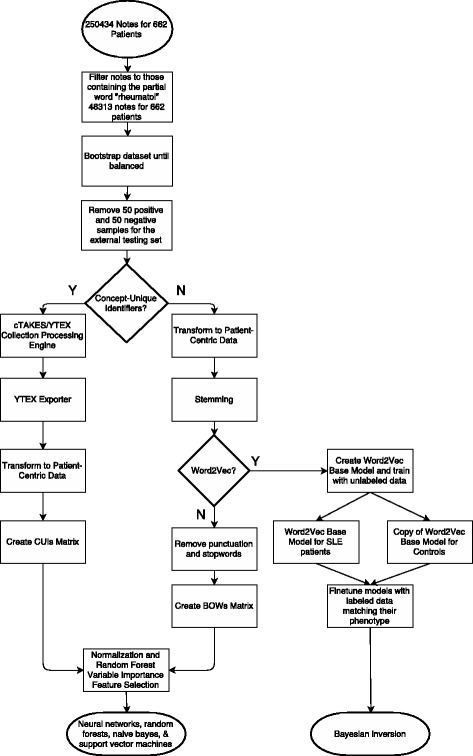
Data Flow and Feature Engineering Pipeline This pipeline shows the flow of data through our clinical pipeline. This pipeline shows how the data is bootstrapped, subsetted and filtered in order to obtain higher quality notes for use in the proceeding feature selection and classification. If CUIs are to be utilized, then the cTAKES/YTEX pipeline is used to create the initial features through usage of the Collection Processing Engine within the cTAKES suite. This data is output as a sparse matrix which we convert in order to conform to the style of the other feature engineering techniques so each algorithm can be used independently of the data it is being given. If CUIs are not being used, then a stemming process is undergone as this is needed for both BOWs and the inversion method. In the case of using BOWs, punctuation and stop words are additionally removed in order to reduce bias in the dataset. If the inversion method is to be used, then we leverage Word2Vec to create two Word2Vec models which are fine-tuned according to which phenotype they represent. All feature sets are subjected to normalization and feature selection through scikit-learn’s ExtraTreesClassifier’s variable importance to prep for classifier usage [22]

The data resulting from the BOW and CUI pipelines were normalized and processed through scikit-learn’s ExtraTreesClassifier using a random forest classifier [22] in order to extract variable importance and reduce features, through usage of the gini index and a feature subset to 100. The resulting matrices were subjected to five different classifiers: neural networks, random forests, naïve Bayes, support vector machines, and Bayesian inversion (Fig. 1). Bayesian inversion is conducted by using separate distributed language representations for each label, or phenotype, in a dataset and using the higher prior probabilities for determining the class label [4]. Here this is implemented with Word2Vec.

#### Neural networks

Both BOW and CUI data were passed through a shallow standard multi-layer perceptron neural network (1 layer of 250 nodes). The neural networks were implemented using the Theano Python library [23–25].

#### Random forests

The random forest algorithm has been used effectively with BOWs and other high-dimensional data in the past [26]. This algorithm was applied to both BOW and CUI data in our experiment. Random forest was implemented using scikit-learn, a Python library which supplies customizable machine learning techniques [22].

#### Naïve Bayes

Naïve Bayes was chosen as a baseline classifier for the NLP output, and was applied to both BOW and CUI pipeline data [27]. Naïve Bayes was also implemented using scikit-learn [22].

#### Support vector machines

Support vector machines (SVM) were selected because they are not prone to error with high-dimensional datasets and have been previously shown to be useful in text-based classification problems [28]. Support Vector Machines were also implemented using scikit-learn [22].

#### Word2Vec Bayesian inversion

Previous work with Word2Vec for text classification focuses mostly on averaging the values of all sentences in a document for use in ML [29]. In departure from this typical method, this experiment created a training corpus for each of the lupus and control phenotypes. As seen in Fig. 1, each corpus started with an unlabeled Word2Vec model based upon all of the training data and was then fine-tuned by adding the SLE patients to one corpus and the controls to the other. This effectively created two neural networks one for SLE-positive patients and one for SLE-negative patients [4, 19]. Each neural network was highly specialized for their respective training corpus. The testing step for this algorithm broke up a patient’s notes by sentences and sentence-by-sentence predicted the log-likelihood that the sentence belonged in each corpus. With two networks for prediction, each sentence has two probabilities associated with it, one for each label. Whichever probability is highest is the label assigned to that sentence. Once this is done for all the sentences for a single subject, then whichever label is the most represented is the label assigned. The parameters utilized with the Word2Vec model were built-in dimension reduction to 500 features, context window of 5, and a minimum sentence word count of 20.

#### Evaluation

Each of the above classifiers was subjected to 5-fold cross validation (CV) repeated 20 times in order to select our models and generate AUCs. When conducting our feature selection for each NLP/algorithm combination, the variable importance feature selection was only fitted on the training data for the current CV fold. The validation set within each fold and the testing set were not used as part of the fitting process. 5-fold cross validation was performed 20 times on the training/validation sets for each algorithm in order to generate the cross-validated accuracy and AUC estimate and the 95% confidence intervals [30]. The cvAUC package in R along with a wrapper script were used to produce the AUCs and confidence intervals [31, 32]. The ICD-9 codes classification was rule-based, hence did not require training or confidence intervals, i.e. patients with an SLE ICD-9 code (of 710.0) were classified as SLE. All classifiers were evaluated using the test data set.

The test dataset is evaluated for each NLP classifier. The classifiers used the 5-fold cross validation results in order to optimize parameters on the validation set associated with its fold. That trained model is then used in to classify the test set.

## Results

The algorithms were each tested with a varying set of parameters in order to tune the algorithms. The neural network performed best with one hidden layer of 250 nodes. The testing varied between 1 and 5 hidden layers with the nodes per layer varying from 10 to 1000. The implemented random forest performed the best with the with 300 trees in the forest (100 and 500 were also tested). Support vector machines worked best with a linear kernel, having also tested radial basis function, polynomial, and sigmoid kernels.

**Fig. 2:**
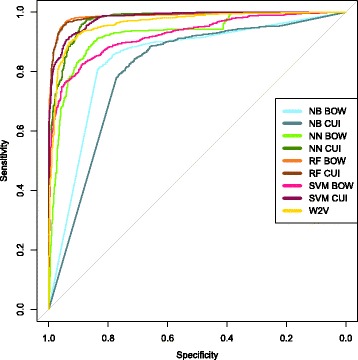
External AUC Curves This graph depicts the AUC of each technique as it performed on the external testing set, generated using the pROC package [42]

The results produced from these aforementioned methodologies are as follows: The algorithms with the best AUC and accuracy, shown in Tables 2 and 3 are neural networks with CUIs, random forests with BOWs and CUIs, and the Word2Vec Bayesian inversion. The test AUCs can also be examined in Fig. 2. ICD-9 codes as a sole metric performed better than expected in the original hypothesis. Additionally, the ICD-9 billing codes and Word2Vec Inversion had similar accuracies and AUCs. The naïve Bayes algorithm performed poorly, as expected. Last, the Support Vector Machines did not provide as accurate results as hoped, but generated a promising AUC with CUIs. Additionally, we performed a permutation/randomization test with our random forest classifier in order to show that our results are well outside the 99% confidence interval for AUC scores resulting from random chance. The distribution of AUCs built through the scrambling of labels in the original dataset resulted in a mean AUC of 0.500 with a confidence interval of (0.499, 0.501) at the 95% confidence level and a mean AUC of 0.500 with a confidence interval of (0.498, 0.501) at the 99% confidence level.

**Table 2:** Table showing each machine learning technique and its 5-fold Cross-Validation accuracy and its test set accuracy with each NLP classifier

Technique	Data form	CV Acc.	CV CI (*α*=0.95)	Test Acc.
ICD-9 billing codes	N/A	89.655	N/A	90.00
Word2Vec inversion	N/A	89.653	[89.281, 90.025]	90.039
Neural network	BOWs	84.138	[80.887, 87.630]	87.100
	CUIs	94.138	[89.539, 92.358]	92.10
Random forests	BOWs	95.172	[93.875, 94.539]	95.250
	CUIs	95.345	[94.889, 95.318]	95.00
Naïve Bayes	BOWs	85.000	[80.141, 83.859]	82.000
	CUIs	81.207	[76.087, 79.013]	77.55
Support vector machines	BOWs	86.724	[83.031, 86.469]	84.750
	CUIs	90.862	[90.470, 92.230]	91.35

**Table 3:** Table showing each machine learning technique and its AUC from the test set and its respective 20x repeated Cross-Validation AUC and confidence interval with each NLP classifier

Technique	Data form	CV AUC	CV CI (*α*=0.95)	Test AUC
ICD-9 billing codes	N/A	0.897	N/A	0.900
Word2Vec inversion	N/A	0.963	[0.956, 0.971]	0.905
Neural network	BOWs	0.902	[0.897, 0.908]	0.925
	CUIs	0.960	[0.957, 0.964]	0.974
Random forests	BOWs	0.981	[0.979, 0.984]	0.987
	CUIs	0.987	[0.985,0.989]	0.988
Naïve Bayes	BOWs	0.841	[0.815, 0.868]	0.841
	CUIs	0.805	[0.777, 0.833]	0.805
Support vector machines	BOWs	0.923	[0.911, 0.934]	0.923
	CUIs	0.980	[0.975, 0.985]	0.980

## Discussion

### Data quality

BOWs’ nature of being error-prone and curation needs were reflected in our results. During our initial study with BOWs, some of the top words for classification were capturing writing styles by SLE researchers, as well as identification numbers for these researchers. This was an egregious problem because these researchers were working with confirmed SLE patients and classifying based off of this would mean that the results were not generalizable to a more general population. We manually removed a large portion these identifiers, styles, and location-based terms and our top words reflected a more general series of words which partially aligned with our top CUIs, as evidenced in Tables 4 and 5. Also, evidenced in the tables are how some artifacts still remained after multiple iterations, such as “843identificationremov” which was used as an early attempt at de-identifying the data.

**Table 4:** Top 25 word stems for BOWs according to the variable importance extracted from scikit-learn’s ExtraTreesClassifier and stemmed using nltk’s SnowballStemmer [17, 22]

Rank	Word	VIMP
1	C3	0.0311
2	Sle	0.0225
3	Graviti	0.0172
4	Sole	0.0126
5	Phurin	0.0113
6	Epitheli	0.0084
7	C4	0.0065
8	Yet	0.0065
9	Lymph	0.0063
10	Hemlymph	0.0059
11	Educ	0.0059
12	Resolv	0.0054
13	912	0.0054
14	Fatigu	0.0050
15	Thrombocytopenia	0.0047
16	2500	0.0047
17	Need	0.0047
18	Naugl	0.0047
19	Clot	0.0043
20	Screen	0.0042
21	Antidoubl	0.0040
22	Beat	0.0040
23	Acut	0.0038
24	843identificationremov	0.0038
25	Pregnanc	0.0036

**Fig. 3:**
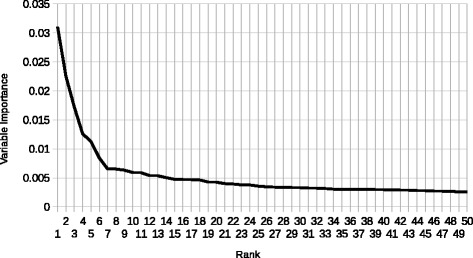
Variable Importance by Word Stem This graph depicts the degradation of variable importance from the top 50 word stems

A graph of the degradation of variable importance for these word stems can be found in Fig. 3

**Table 5:** Top 25 CUIs according to the variable importance extracted from scikit-learn’s ExtraTreesClassifier [22]

Rank	CUI	Description	VIMP
1	C0042014	Laboratory: Urine Examination	0.0307
2	C0699177	Plaquenil	0.0258
3	C0024141	Systemic Lupus Erythematosus	0.0236
4	C0194073	Kidney Biopsy	0.0208
5	C0024204	Lymph Node	0.0179
6	C0008031	Nonspecific Chest Pain	0.0166
7	C0018966	Heme	0.0158
8	C2711450	Enlargement (Morphological Anomaly)	0.01502
9	C0014597	Epithelial Cell	0.0111
10	C0023516	Leukocytes	0.0100
11	C0003243	Antinuclear Antibody (ANA)	0.0094
12	C0002170	Alopecia	0.0089
13	C0024202	Lymph	0.0085
14	C1267547	Entire Mouth Region	0.0084
15	C0009780	Connective Tissue	0.0083
16	C0229671	Serum	0.0068
17	C0042036	Urine	0.0065
18	C0014060	St. Louis Encephalitis	0.0062
19	C0038999	Swelling	0.0061
20	C1269549	Entire Zygoma	0.0060
21	C0036749	Serositis	0.0060
22	C0033684	Proteins	0.0059
23	C0014239	Endoplasmic Reticulum	0.0059
24	C0009782	Connective Tissue Disorder	0.0058
25	C0024143	Lupus Nephritis	0.0058

**Fig. 4:**
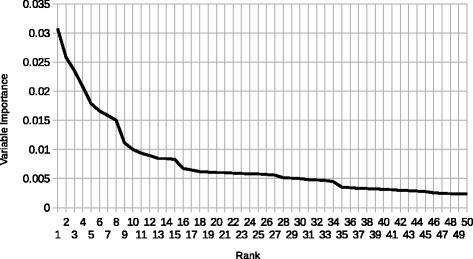
Variable Importance by CUI This graph depicts the degradation of variable importance from the top 50 CUIs

CUI descriptions were extracted from MetamorphoSys [7]. A graph of the degradation of variable importance for these CUIs can be found in Fig. 4

We hoped the CUIs seen in Table 5 would contain a subset of ACR criteria 1 that are not lab test specific. The only CUI directly discovered in our feature selection is C0003243, which is ANA, criteria 11 in the list of ACR criteria seen in Table 1. What is unknown however is whether positive antinuclear antibody or negative antinuclear antibody is what is being found as it is not believed that cTAKES/YTEX’s negex would function as negative counts for negative antinuclear antibody and positive counts for positive antinuclear antibody. Lupus nephritis, as indicated by SLICC to be indicative of a positive SLE diagnosis in the presence of positive ANA, was the 25th most important CUI according to Table 5, showing that CUIs are capturing data pertinent to the classification of SLE [15]. Additionally, the table revealed further data quality issues when considering other diseases common to the Rheumatology Clinic as CUIs for both Rheumatoid Arthritis and History of Rheumatoid Arthritis were present, creating a confounding problem when considering the classification of SLE. This becomes problematic in classification as Rheumatoid Arthritis (RA) and SLE share an amalgam of symptoms. It is worth noting that there exists a CUI for lupus erythematosus in addition to the CUI for SLE, but the CUI for lupus erythematosus was not flagged in our clinical text, while the CUI for SLE appeared repeatedly. Other issues noted with the CUI approach are misinterpreted acronyms and abbreviations; for example, “SLE” seems to have been misinterpreted in some instances as St. Louis Encephalitis. This is not surprising since “SLE” appears as a synonym in some of the UMLS ontologies for CUI C0014060, which stands for St. Louis Encephalitis. Similarly, “ER” appears as a synonym for C0014239 the CUI for Endoplasmic Reticulum in the UMLS. “ER” has been used frequently in the rheumatology clinical notes in the context of Emergency Room visits. As for “Heme”, the term is sometimes used as abbreviation for hematology, and lupus patients often have hematological abnormalities. Similarly, “enlargement” is mentioned repeatedly in clinician’s notes in reference to lymph nodes or spleen enlargements, which are common findings in lupus.

### Classification

ICD-9 billing codes have been proven to be ineffective classifiers in the past [1–3]. Table 2 shows that ICD-9 reached an AUC of 0.897 with an 89.655% accuracy on the cross validated set. On the external test set, ICD-9 performed, effectively, the same, reaching a 90% accuracy and a 0.900 AUC. Predescribed, ICD-9 codes’ error rates were lowest at 17.1%, but the results show a lower error rate than this. It is believed that this lower error rate is due to the limited number of representative ICD-9 codes used for SLE at MUSC. The AUC and accuracy for the ICD-9 billing code classification have similar values because there is no probability in determining SLE status from an ICD-9 code. This is because of the pre-described method for determining whether or not a patient has SLE: If a patient had at least one mention of the SLE ICD-9, then they were classified as having it.

We also utilized the naïve Bayes classifier as a baseline algorithm alongside the ICD-9 billing codes. One of the main assumptions for the naïve Bayes algorithm is that the features in a dataset are independent [33]. BOWs is inherently independent as feature counts functioning as data forces each feature to act independently. In the past, this has made naïve Bayes a favorable classifier for text categorization [27]. However, it has been seen recently that naïve Bayes is performing worse than other algorithms on these datasets because the sheer amount of training data has favored other algorithms ahead of this now considered baseline [27, 34]. This expected outcome supported our hypothesis as naïve Bayes with BOWs achieved an accuracy of 85.000% on the cross validated dataset, AUC, with 82.000% accuracy on the external testing set, AUC.

BOWs with our dataset had the best success with random forests and text classification has proven to have great success with random forests in the past [35, 36]. This is because the random forests algorithm is able to overcome some of the pitfalls with the BOWs representation of a textual dataset. Random forests can do this because it is an ensemble technique, composed of many decision trees, which takes advantage of how decision trees overfit to training data by bagging a group of trees together and taking the mode classification for each patient. The ensemble does not produce the same tree every time because each tree in the ensemble makes its decision based upon a subset of features [37].

CUIs with our dataset had the best success with neural networks, which have been historically proven to work well with textual datasets [38]. It is worth noting that the neural network we used only contained one hidden layer, so the network is considered to be very shallow. Deep neural networks are the typical use case for finding hidden patterns within data for purposes of classification [39–41]. However, our results suggested that deep neural networks were not viable for either BOWs or CUIs for this dataset. We believe this is because our dataset is not large enough for deep neural networks to discern hidden patterns within data. Shallow neural networks, on the other hand, proved to be exceedingly viable for predicting SLE with CUIs as the input. This is because rather than having the neural network find all the patterns in a dataset, CUIs contain intrinsic patterns so the neural network does not have to discover all the patterns by itself, but, rather, find patterns using these designed concepts.

Chosen because of proven success with non-clinical datasets [4], Word2Vec Bayesian inversion proved to be an effective classifier for this data. We are the first to try this with clinical EHR data. Because the neural networks that make up this inversion algorithm are embedded into Word2Vec itself, we cannot peer into the system to extract which words are more indicative of this corpora ensemble classification. BOWs were seen as having multiple iterations of steps to remove bias from the textual dataset and extra time usage in removing or substituting punctuation, which removes a lot of generalizable classification power unless this is consistently done. This detriment is very undesirable as it leaves BOWs to only be useful in a very specific context; here, BOWs would only be useful within MUSC without this iterative removing, as mentioned in the Data Quality section. CUIs were seen as having a large overhead in their creation, a heavy detriment on time. This Bayesian inversion, however, does not need any form of time-consuming or error-prone preliminary steps as the inversion method only underwent stemming, as evidenced in Fig. 1. Additionally, Word2Vec’s usage of contexts around words creates a more robust model which can adapt to any dataset. This inversion classifier is also adaptable to classifications that are not strictly binary. When creating specific corpora for this dataset, one Word2Vec model learns what is indicative of SLE through word probabilities while the other model learns what is indicative of non-SLE through word probabilities. This is easily expanded if, for example, we wanted to add another phenotype to this classification task. With labeled data for RA, for example, three models could be made: one for SLE, one for RA, and one for controls. Manual curation of the data outside of labeling would not be needed to add the RA data to the dataset for the inversion method since the inversion uses its internal neural network in order to create and learn its own features without any user input or curation. We only focused on the results of the inversion method for the word vectors as the word averaging technique produced poor results in early empirical stages. The Word2Vec model can be tuned in multiple ways as there are several variables to fine-tune, as outlined in the methods: feature set size, context window, and sentence word count.

### Future work

In its current state, our pipeline is performing very well with the large amount of clinical notes used. The results could be more refined, however, by breaking the notes for each patient down into document zones and using this data to better classify patients. For example, a family history section may influence a classifier when the data coming in at that point was only referring to a family member. This data is still important as if there is any genetic component (or analogous component for other document zones), then that data is still valuable and can be used in the classifier.

Additionally, lab tests, as described in the ACR criteria (Table 1) engineered into their own features would only serve to aid in classifiers, at least in the neural network and random forests cases, as they are clearly indicative of diagnoses in some cases [15].

There exist several improvements we suggest to the usage of this Word2Vec Inversion method. In its current state, the inversion method treats every sentence as an equal weighting into learning specialized corpora. While we attempted to filter to only relevant notes with the “rheumatol” filtering, there are still sentences that are being utilized for evaluation which add little to nothing to classification power, while detracting from the classification power of other sentences. The inversion method could also be combined with other methods in order to create more refined classifiers. For example, the probability that a given sentence belongs to a specific label could be simply added as a feature to the BOWs feature vector in order to combine the two algorithms into one.

## Conclusions

In examining the varying NLP classifiers for classifying and processing the SLE patients based on EHR notes, we have determined the relative effectiveness of several different algorithms. Neural net- works with CUIs and random forests with BOWs or CUIs are all equally powerful in classification tasks for unstructured clinical notes, especially when compared to baselines such as utilizing ICD-9 billing codes and a naïve Bayes algorithm. The Word2Vec Bayesian inversion technique is shown to be powerful in the cross-validated results, however the accuracy and AUC in the external set were slightly diminished. Nevertheless, this method has less overhead than that required for generating CUIs, less dependencies and testing than that of BOWs and CUIs, and is more easily scalable beyond binary classification tasks. Word2Vec Bayesian inversion is a promising methodology especially when combined with other classifiers and warrants further research to refine and improve its output.

## Abbreviations

ACR: American College of Rheumatology
ANA: Antinuclear antibodies
AUC: Area under the receiver operating characteristic curve
BOWs: Bag-of-Words
cTAKES: Clinical text analysis and knowledge extraction system
CUIs: Concept unique identifiers
CV: Cross-validated
EHR: Electronic health record
ICD-9: International classification of diseases, ninth revision
ML: Machine learning
MUSC: Medical University of South Carolina
NLP: Natural language processing
RA: Rheumatoid arthritis
ROC: Receiver operating characteristic
SLE: Systemic lupus erythematosus
SLICC: Systemic lupus collaborating clinics
SVM: Support vector machines
UMLS: Unified medical language system
YTEX: Yale cTAKES extension for document classification
